# A Fatal Alliance between Microglia, Inflammasomes, and Central Pain

**DOI:** 10.3390/ijms21113764

**Published:** 2020-05-26

**Authors:** Stefanie Hoffmann, Cordian Beyer

**Affiliations:** 1 Department of Psychiatry, Psychotherapy and Psychosomatics, University Clinic, RWTH Aachen University, 52074 Aachen, Germany; sthoffmann@ukaachen.de; 2 Institute of Neuroanatomy, RWTH Aachen University, 52074 Aachen, Germany

**Keywords:** microglia, neuroinflammation, inflammasome, NLRP, pain

## Abstract

Microglia are the resident immune cells in the CNS, which survey the brain parenchyma for pathogens, initiate inflammatory responses, secrete inflammatory mediators, and phagocyte debris. Besides, they play a role in the regulation of brain ion homeostasis and in pruning synaptic contacts and thereby modulating neural networks. More recent work shows that microglia are embedded in brain response related to stress phenomena, the development of major depressive disorders, and pain-associated neural processing. The microglia phenotype varies between activated-toxic-neuroinflammatory to non-activated-protective-tissue remodeling, depending on the challenges and regulatory signals. Increased inflammatory reactions result from brain damage, such as stroke, encephalitis, as well as chronic dysfunctions, including stress and pain. The dimension of damage/toxic stimuli defines the amplitude of inflammation, ranging from an on-off event to low but continuous simmering to uncontrollable. Pain, either acute or chronic, involves inflammasome activation at the point of origin, the different relay stations, and the sensory and processing cortical areas. This short review aimed at identifying a sinister role of the microglia-inflammasome platform for the development and perpetuation of acute and chronic central pain and its association with changes in CNS physiology.

## 1. Microglia—A Genuine All-Rounder

Microglial cells originate from primitive yolk sac progenitors and colonize the mammalian brain early during development [1]. A battery of different transcription factors/cytokines is involved in their development and differentiation [2]. Microglia are scattered throughout the brain but appear heterogeneous with respect to their distribution, morphology, and functionality [3], thus forming a multitasking cell population with functional specializations. During development, microglia play an active role in the development, plasticity, maintenance, and pruning of synapses [4]. This enables microglia to influence nerve cell survival and to shape neural networks. In the adult brain, they fulfill several immunological functions. Microglia can be attributed a guard function by continuously surveying the brain parenchyma for pathogens. They phagocyte debris, initiate immune responses, secrete inflammatory mediators, and communicate with other immune-relevant cells, either brain-intrinsic or invaded from the peripheral circulation [5,6]. Besides, this cell type possesses several other functions, including the control of brain ion homeostasis, i.e., calcium, and growth factor supply, i.e., insulin-like growth factor-1 (IGF-1). For more information on the multifaceted functions of microglia in the developing and adult brain, we referred to the review article of Li and Barres [7].

Classification of adult microglia with so-called “classically activated” pro-inflammatory M1 or “alternatively activated” anti-inflammatory M2 cell forms has been made on the basis of functional and phenotypic appearance [5,6]. Microglia of the M1/2 type, most likely, do not represent different cell populations rather than a single cell population switching from one state into the other and backward depending on the regulatory signals. In the past few years, this simple categorization was further refined with additional intermediate stages, requiring a categorization on the basis of their functional properties. Stratoulaias and collaborators [8] recently presented a convincing grading of microglia according to their phenotype and function, which meets the demands of assigning microglia to genomic, spatial, morphological, and physiological properties. In particular, they proposed a categorization, which includes aspects, such as developmental, homeostatic, immunological, and disease-associated functions, and related these characteristics to the expression of selective expression markers.

From a functional point of view, microglia principally serve as the resident immune cell within the brain. Due to their high motility, they continuously survey distinct districts of brain parenchyma for any pathological changes and respond to acute and chronic injuries and infections by dynamically extending and retracting their processes [5,6]. Toxic and danger signals contribute to concerted action to the activation of microglia. This entails their switch in morphological appearance from a ramified to a more rounded cell type, which also reflects associated changes of functionality [5,6]. Battling activated microglial cells rapidly migrate to and accumulate at the injured site, proliferate, i.e., microgliosis, and become phagocytic active. However, the phagocytosis function can be sometimes disturbed under distinct pathological conditions. Heneka and collaborators demonstrated, for instance, that norepinephrine (NE) suppressed amyloid-ß-induced activation of microglia and that NE-depleted amyloid precursor protein (APP)-transgenic mice revealed impaired microglia phagocytic activity [9]. Upon activation, they start communicating by soluble factors and cell–cell contacts with local neurons and, in particular, astrocytes, the latter then typically becoming activated and proliferate, i.e., astrogliosis [10,11]. Finally, microglia, astroglia, neurons, and endothelial cells form an inflammatory network to initiate early inflammatory responses (see next paragraph), coordinate other inflammatory “team players”, the opening of the blood-brain barrier (BBB), and invasion of peripheral immune cells [12,13,14].

Depending on their functional state, microglia can either cause neurotoxicity or promote neuroregeneration and repair while limiting secondary inflammatory-mediated damage [15]. The latter also includes the promotion of corralling and wound compaction after spinal cord injury [16] and vascular reorganization and the maintenance of physiological homeostasis [17].

A novel interesting observation regarding microglial immune-related functions is that these cells experience a so-called “sexual differentiation” in their function and physiology, which links them to a sexual bias in the susceptibility and severity to neurological diseases and existing gender differences of brain disorders [18]. In line with this are findings that microglia express a variety of receptors of gonadal steroid hormones and even change their steroid receptor profiles along with toxic and degenerative disease models [19,20].

All in all, there is increasing evidence that microglial cells can be attributed a number of different physiological functions in the unaffected differentiating and adult brain. These include (i) the modulation of neural networks by controlling the synaptic communication (i.e., synapse elimination or stabilization), thereby guaranteeing neuronal survival within such networks, (ii) calcium homeostasis and growth factor supply, thereby promoting nerve cell functionality, (iii) the laminar orientation of neurons, (iv) vascularization, and (v) myelination [7]. Thus, microglial cells are indispensable for regular brain development and adult brain function. The phagocytic and immune-related actions of microglia and brain-invaded macrophages are unique characteristics within the adult CNS and essential for brain-intrinsic immune responses after acute brain trauma or sterile pathological processes [7]. The next chapter contains a brief overview of the inflammatory role of microglia with a special emphasis on inflammasomes.

## 2. Inflammasomes—No Way around When Talking about Inflammation

Inflammasomes comprise a key cytoplasmic multiprotein complex that sense damaging-toxic conditions and initiate the set-off and boost of the inflammatory cascade, in particular IL1β and IL18 production. First described in the early millennium and attributed to phagocytic leukocytes [21,22], the expression of inflammasomes is now assured for a large variety of cells, including neural cells being implicated in the immunological scenario in some way or another [23,24]. Structurally, inflammasomes are assembled from three different main molecular units, including a cytosolic sensor (nucleotide-binding domain leucine-rich repeats, NLR), an adaptor protein (apoptosis-associated speck-like protein, ASC), and an effector caspase (caspase-1, Casp1) [23,24,25]. ASC has two domains—a Casp1 activation and recruitment domain—which interacts with the pro-Casp1, and a pyrin domain, which binds to NLR. Upon complete assembly, the released active Casp1 cleaves the precursors of IL1β and IL18, as well as the pyroptosis inducer gasdermin D into active peptides by proteolytic degradation. The activation of the NLR inflammasomes is perhaps best illustrated by the two-step model [26]. During a priming step, pro-IL1β mRNA is transcribed, and the mRNA translated into the corresponding protein. A second activation step then involves the recruitment and activation of Casp1 by inflammasome assembly. Finally, the transformation of the pro-IL1β into its mature form takes place.

The activation of inflammasomes occurs through recognition of specific inflammatory ligands called pathogen-associated molecular patterns (PAMPs, i.e., pathogen-specific carbohydrates and lipoproteins, such as lipopolysaccharides (LPS), or nucleic acids) and damage-associated molecular patterns (DAMPs). The latter are derived from host cells, such as tumor cells, dying cells, or products released from cells in response to toxic signals and traumatic processes and induce sterile inflammatory responses [27]. PAMPs and DAMPs bind to pattern recognition receptors (PPRs), which include Toll-like receptors (TLR), cytoplasmic NOD-like receptors (NLR), intracellular retinoic acid-inducible gene-I-like receptors, transmembrane C-type lectin receptors, and absent in melanoma 2-like receptors (AIM2) [28,29]. DAMPs typically bind to cytosolic receptors, whereas PAMPs signal through membrane receptors. Irrespective of the signal perception, the activation of the intracellular inflammasome complex is believed to represent the first executive downstream platform of both PPRs [30]. The NLR receptor superfamily is a heterogeneous protein family of cytoplasmic immune receptors consisting, to date, of fourteen family members (NACHT, LRR, and PYD domains-containing protein, NLRP1-14). Besides extracellular-regulated inflammasome activation, intracellular pathophysiological processes, such as increased reactive oxygen species (ROS) formation, mitochondrial dysfunction, and collapse, as well as nuclear damaging with free DNA molecules, also contribute to the final activation of inflammasomes [31]. The inflammasome platform and microglia constitute important arms of the innate immunity in the CNS and represent a decisive primary line of the sterile or nonsterile host defense system [28,32].

A number of studies have shown that the CNS is fully equipped with different inflammasome subtypes, although the expression profiles vary among neural cell types and brain regions as well as in response to the damaging stimuli [23,24,25]. Inflammasomes, such as NLRP3, NLRP1b, and the NLR family CARD domain-containing protein 4 (NLRC4), appear to be mainly expressed in the brain under various pathological conditions, ranging from acute trauma, such as stroke and spinal cord injury, to chronic neurodegeneration, including amyotrophic lateral sclerosis (ALS), multiple sclerosis (MS), Alzheimer’s disease (AD), psychiatric disorders, including depression and others [23,24,25,33,34,35]. A number of studies in the last decade have collectively identified mainly the NLRP3 inflammasome as the dominant driver of autoimmune processes, acute damage, and tissue inflammation in the CNS [24,25,29,36,37,38].

From a functional point of view, it seems clear that microglia as the major brain-resident immune cell is the source of inflammasomes. This is the case particularly under post-injury conditions after acute trauma but also evident in chronic disease models, such as AD and major depressive disorders (MDD) [37,38]. Notwithstanding the overall importance of the inflammasome-microglia assignment, other neural cells (astroglia, neurons) also express inflammasomes under specific pathological circumstances [25,34,35].

In the following chapter, we have followed in more detail the questions (i) whether inflammasomes become activated during pain and (ii) which role can be attributed to microglia under such dysfunctional conditions?

## 3. Pain in the Brain—Aching Microglia

Pain, in general, represents a neural and immune-related defense system that responds rapidly to harmful internal and external stimuli through the ascending somatosensory neuronal pathway. According to textbook classification [39], pain is usually transitory until noxious stimuli are resolved, or the underlying tissue damage or pathology has cured. However, painful conditions as a consequence of arthritis, peripheral neuropathies, cancer, etc. can persist for longer even years, leading to chronic pain symptoms [40]. Pain can thus be classified as acute or chronic-persistent even when the noxious, toxic irritation is removed in certain cases [41]. In certain cases, pain is a vegetative-cognitive perception in the absence of any detectable harmful stimuli, disease, or tissue injury and is often accompanied by psychiatric disorders [42]. Irrespective of the trigger and the form, the perception and transmission of pain use well-known anatomical sensors, fiber tracts, and trails, usually beginning in the organ/tissue and continuing through the spinal ganglion, the dorsal horn of the spinal cord, ascending spinal tracts, thalamus, and then spreading to relevant cerebral cortex areas where the pain is first brought to mind within the somatosensory regions and further processed, analyzed, modulated, and valued by other secondary cortical and subcortical centers [43,44,45]. We did not intend to discuss here the routes of pain to the brain and their relay points on the way. This is a very complex issue and often reviewed by others and mentioned in textbooks (see textbook reference above). Rather, we wanted to pinpoint the involvement of microglia and inflammasomes in this process [41].

Functional brain imaging studies in humans have shown that acute pain evoked by nociceptive stimuli is typically accompanied by the activation of a widely distributed network of cerebral cortex structures, i.e., subcortical areas, including the thalamus, somatosensory, insular, and anterior cingulate cortices [46]. This indicates the presence of a highly conserved core set of pain-related brain centers and various switchboards related to central pain transmission irrespective of the spinal dorsal horn and the ascending pain fiber tracts. First, we have inquired about the input of local central microglia to pain signaling. Neuro-immune interactions involving local cerebral cortex microglia appear to play a critical role in the generation and maintenance of neuropathic pain [47]. Microglia produce a set of nociception-related mediators (cytokines, growth factors), thereby altering nociceptive signaling cascades (NF-κB, JAK/STAT, MAPK, PI3K/AKT) in the brain [47,48,49]. Importantly, microglial P2 × 4/P2RX7 receptors act a core microglia-neuron signal system in pain regulation [49,50]. Similar to the cerebral cortex, microglia in the thalamus and spinal cord is likewise implicated in pain transmission and manifestation [51,52,53], thus highlighting the complexity of the modulation of ascending pain fiber pathways and by local microglia.

### 3.1. Spinal Cord—Pain—Microglia

What is known about the role of microglial for pain perception, modulation, and transmission in the spinal cord? Neuropathic pain and other pain-related stimuli induce strong microglia proliferation and activation in different animal models (reviewed by [53]). Upon stimulation, microglia produce a battery of pain mediators, including ILs, chemokines, prostaglandin E2 (PGE_2_), and many more. The tetracycline minocycline, which selectively inhibits pro-inflammatory M1 polarization and expression of M1 markers [54], described as a microglia inhibitor attenuated neuropathic pain resulting from peripheral nerve lesions only at early times, suggesting a potential role for this cell type in the origin of pain mechanisms [55]. The purinergic receptor P2X7 is expressed in microglia, and ATP as a ligand appears to play an important role for microglia activation under such circumstances [56]. This is valuable information since P2X7 is also well-known to be involved in the rapid assembly and activation of inflammasomes [21,24]. TLRs are equally important for inflammasome regulation and are implicated in microglia-dependent spinal cord pain sensitization [57], which requires the induction of mitogen-activated protein kinases (MAPK) [58]. This highlights the crucial role of spinal inflammasomes in pain transmission. Recent findings support this assumption by providing evidence that the inflammasome NLRP3 is significantly activated in spinal cord microglia under neuropathic pain [59,60]. Since opioids are important for pain management, the relationship between opioids, microglia, and inflammasomes is of interest. This also concerns interactions between the dorsal horn, pain gating, and stress/psychiatric disorders. In general, there is only sparse information using literature surveys. However, it has been found that morphine induces and prolongs neuropathic pain by increasing spinal microglial NLRP3 activation and inflammatory responses [59,61]. Second, a medulla-spinal cord circuit composed of descending GABAergic tracts controls spinal pain thresholds by modulating spinal encephalin/GABAergic interneurons. Stress clearly disturbs this descending pain-modulatory pathway, thereby reducing pain thresholds [62]. In line with this, rats with genetically predisposed depressive behavior have revealed lower nociceptive thresholds involving the central melatonin system and NMDA receptor modulation [63]. The latter observation highlights the comorbidity between pain and depression.

In a synopsis, local spinal microglia can powerfully regulate pain onset and intensity by producing a set of pain-related pro-inflammatory mediators involving inflammasome activation [59,60,64]. It is also of importance that spinal hyper-activation of microglia often persists weeks after the original pain induction, thus representing a sensitized local cellular network causing a low-scale pro-inflammatory situation, which keeps pain mechanisms running [64].

### 3.2. Thalamus—Pain-Microglia

The next important control entity and relay station, transmitting pain, is the thalamus. Nociceptive neurons of the spinal cord and the thalamus communicate pain-related information in terms of calibrated firing patterns through the spinothalamic tract. These interactions appear to be balanced and influenced by local spinal and thalamic glial cells [50]. Rostral to the spinal responses, microglial activation, demonstrated by pro-inflammatory cytokine profiles, is observed in a sequential way first in the spinal cord, followed by corresponding thalamic relay nuclei, such as the venter-posterior lateral (VPL) and venter-posterior medial (VPM) thalamic nuclei [65,66]. These glial responses then modulate the related neuronal networks [50]. The use of positron emission tomography (PET) and radiolabeled ligands for the benzodiazepine receptor, i.e., a marker of microglia activation, in patients suffering from phantom limb pain has also revealed strong microgliosis in the thalamus [67]. To our surprise, very few publications have ever studied inflammasome expression in the thalamus. In our research group, we could demonstrate that motoneuron degeneration in a rat ALS model (SOD1(G93A)) triggers the expression of inflammasomes in thalamic nuclei. We found neurons and astroglia fully equipped with NLRP3, ASC, and IL1ß with NLRP3, whereas microglia only revealed ASC [68]. It is not clear at present why microglia do not contain the full set of inflammasome components. One explanation could be that the degenerative stimuli are not sufficient to trigger a full response in microglia. More pain-related, Li and colleagues [69] presented a meta-analysis, showing that the NLRP3 inflammasome plays a prominent role in post-stroke pain processing, and concluded that NLRP3 activation in thalamic and cortical microglia accounted for GABAergic alterations and thalamic lesions. Although microglial activation in the thalamus is clearly associated with chronic and acute pain transmission, the data situation about the thalamus and inflammasomes in pain signaling is still vague.

### 3.3. Sensory Cortex and Associated Areas—Pain-Microglia

We further moved on and discussed data obtained from the sensory cerebral cortex and related higher brain centers, the central destination of ascending pain signals, and associated pain centers, additionally receiving thalamic input [70]. Thalamic projections arising from the lateral part synapse on the primary somatosensory cortex are responsible for the immediate awareness of a painful sensation and the exact location of the painful stimulus, thus representing the sensory-discriminative component [71,72]. Thalamus-cortical tracts having their origin in the medial parts reach different limbic areas, including the anterior cingulate cortex and insular cortex, and code emotional-affective qualities of pain [73]. It is also well-accepted that maladaptation of the thalamus-cortical projections can lead to chronic pain [73]. Neuroinflammatory processes occur, as shown in the previous parts of the article at all places, where nociception is generated and transmitted. It is also evident that activated microglia, at least in the spinal cord under acute pathophysiological conditions, is involved, if not even the pivotal driving force, for pain generation [70]. Increased microglial activation has also been described in higher brain centers, including the periaqueductal gray and hypothalamus after peripheral nerve damage in rats [74]. PET studies using ligands for the translocator protein (TSPO), a marker for activated glia, in human patients with back pain have further demonstrated increased labeling of the putative somatosensory cortical representation of the lumbar region [75]. This is supported by data showing microglia activation in the sensory cortex and amygdala of rats under chronic pain conditions [76]. Generally, neurogenic inflammation and pain sensation associated with migraine in humans leads to a cortical spreading of labeling of the peripheral benzodiazepine receptor known as a microglia marker for activation by using the PET ligand ^11^C-PK11195 [77]. From the data, we have presented, so far, we might conclude that microglial cells are involved in the modulation of pain processing at different neuroanatomical sites. It is, however, not clear to which extent these cells are implicated in the initiation and perpetuation of nociceptive responses. In the following, we have put emphasis on the aspect of whether microglial inflammasomes are embedded in pain perpetuation.

### 3.4. Brain Pain Centers—Inflammasomes

The question now arises whether inflammasome stimulation in microglia is induced or dysregulated in higher cortical brain centers with respect to the activation of ascending nociceptive signals. Central post-stroke pain resulting mainly from ischemic stroke affects the thalamus-cortical pathway either indirectly by influencing fiber tract activity via affecting thalamic GABAergic signaling or directly by causing thalamus lesions [69]. Ischemic, as well as hemorrhagic stroke, is characterized by long-lasting stimulation of the NLRP3 inflammasome complex in rodent microglia [25,78,79]. Each of the above models is closely associated with microglial NLRP3 activation and ends up in chronic pain [69]. At this stage, it is not clear whether NLRP3 activation and chronic pain origin is a correlative or causative phenomenon. Since under chronic conditions, the acute tissue damage is finished, tissue remodeling has mainly taken place, and acute inflammatory responses are contained, it seems plausible that long-lasting and mild-pro-inflammatory processes initiated by microglia could be indeed causative to keep pain mechanisms running. In general, NLRP3 seems to be mainly responsible for the manifestation of central pain irrespective of the cause and is seen, for instance, in different rodent models, mimicking central post-stroke pain [69], peripherally-induced neuropathic pain [80], migraine [81], hydrogen-rich saline-induced hyperpathia [60], the genesis of trigeminal-induced headache [82], and cingulate cortex allodynia [83]. Chronic stress-induced visceral pain has been found to depend on central TLR4 activation, i.e., a major signaling receptor system for NLRP3 inflammasome activation [84] and the downstream cellular cytokine pain cascade, demonstrating the importance of Casp1-dependent IL1ß formation [85]. This highlights the involvement of the IL1ß/inflammasomes system in the CNS during the chronification of pain. The involvement of the NLRP3 signaling axis for the clinical manifestation of pain is supported by showing that the microRNAs miR-34c/miR-23a, which are known to suppress NLRP3 mRNA translation, inhibit the development of the neuropathic brain in rodents [86,87]. Pathologically altered spinal horn neuron function besides spinal microglia activation in the neuropathic brain also appears to contribute to the manifestation of central pain. Their firing signals convey to the anterior cingulate cortex and are responsible for cortical long-term plasticity, which, in turn, contributes to chronic pain conditions [88].

### 3.5. Pain—Psychiatric Disorders-Microglia-Inflammasomes

Of importance, psychiatric disorders, such as major depressive disorders (MDD) and chronic stress, are often associated with neuroinflammation and typically seen as comorbidities for the development of central pain syndromes [89]. Both pathophysiological scenarios imply the activation of microglia and mutually drive each other to remodel brain circuits and synaptic plasticity, leading to chronic pain manifestation and pain sensitization [89,90]. In the past few years, several review articles have highlighted the importance of microglia during the development and manifestation of psychiatric disorders, in particular for MDD [91,92,93]. This has led to the current working hypothesis that depression might be a microglia disease with microglia representing an interface in the pathology of neurocognitive disorders [93,94]. This is supported by clinical studies, which show that microglial markers in the cerebral cortex are related to cognitive dysfunction in MDD (as an example, the translocator protein total distribution volume, TSPO V_T_, analyzed by PET) [95]. In a mouse model for depression, microglia hyper-ramification occurred in the hippocampal dentate gyrus [96]. This effect, as well as depressive-like behavior, were ameliorated after treatment with the anti-depressive drug venlafaxine. Moreover, CX3CR1-deficient mice were resistant to stress-induced depression and changes in microglia morphology [87]. The future will evidence to what extent and in which subgroups of depressive patients, anti-inflammatory treatment will be a therapeutic option [97]. At least, a clinical pilot study supports the idea of treatment of MDD with anti-inflammatory and microglia-targeting compound minocycline [54,98]. Since microglia-driven neuroinflammation appears to be critically involved in the development and linked to the escalation of psychiatric disorders and pain symptoms, the question, therefore, is inflammasomes play what role during these processes. Wohleb and colleagues created the “inflammasome hypothesis of depression”, which favors the idea that inflammasomes are key actors in the etiopathophysiology of MDD and pain [90,91]. Indeed, the hippocampal NLRP1 pathway is important for the development of the depression-dependent chronic neuropathic brain in a rodent model [98]. In support, P2X7 receptor signaling and Casp1 activation both represent critical steps for inflammasome activation and for central pain manifestation, as well as for the development of depression [99]. Similarly, pain is modulated through NLRP3 activation via kinase-dependent phosphorylation [100], and antidepressants confer positive effects on chronic stress and related pain symptoms via NLRP3 inflammasome regulation [101]. Thus, NLRP3 might serve as an additional therapeutic target in MDD [102]. Another study has reached a similar conclusion and found that Cathepsin B, which is associated with the production and secretion of IL1ß through the processing of procaspase-3 in phagolysosomes of microglia, is implicated in the development of inflammatory brain diseases and linked to depression, stress, and pain [103].

Although we are only at the beginning of understanding the complex relationship between microglia, inflammasomes, and pain and their comorbidity with stress and psychiatric disorders, we see strong cross-connections between these pathologies. Despite this evidence, it is tempting but perhaps premature to put inflammasomes/neuroinflammation in the center of pain, depression, and stress.

## 4. Closing Remarks—Inflammasomes in the Center of Pain Disorders

It is safe to conclude that central inflammasome activation, mainly concerning microglial NLRP3, is a decisive and course-setting event in the development and perpetuation of neurological and psychiatric disorders, regardless of whether they are acute or chronic. At this place, we would like to point to the fact that inflammasomes are also expressed by other cell types (astroglia, neurons) in the CNS, depending on the pathophysiological conditions [24,25,34,35,68]. This complicates, to some extent, the characterization of the microglia-inflammasome system during pain perception, transmission, and chronification. However, we feel that microglia-related inflammasomes play a more pivotal role in the ascending pain system than other CNS cell types.

Central pain can be regarded as an “end-phenomenon” caused by acute peripheral injuries or other pathological events, as well as by chronic conditions of known and unknown etiology. Stress and psychiatric diseases trigger and boost microglia and inflammasomes and are, therefore, inevitably linked to central pain manifestation and processing. This can reveal new ways of understanding and clinical handling of pain by targeting inflammasomes in microglia. At least for MDD and stress, preclinical and clinical evidence exists, which demonstrates the efficacy of inflammasome targeting for the disease course and severeness [104,105,106,107]. Thus, inhibition of NLRP3 inflammasome pathways has the potential in counteracting central neuroinflammation, metabolic dysregulation, and immune/inflammatory responses in such diseases [108,109,110].

## Abbreviations

Aß	Amyloid-ß
AD	Alzheimer’s disease
AIM2	Absent in melanoma 2
AKT	Protein kinase B
ALS	Amyotrophic lateral sclerosis
APP	Amyloid precursor protein
ASC	Apoptosis-associated speck-like protein containing a caspase recruitment domain
BBB	Blood-brain barrier
CARD	Caspase activation and recruitment domain
Casp1	Caspase 1
CNS	Central nervous system
DAMPs	Damage-associated molecular pattern molecules
IGF-1	Insulin-like growth factor-1
IL	Interleukin
JAK/STAT	Janus kinases/Signal transducer and activator of transcription proteins
LPS	Lipopolysaccharides
MAPK	Mitogen-activated protein kinases
MDD	Major depressive disorder
MS	Multiple sclerosis
NF-κB	Nuclear factor-kappa B
NLRC4	NLR family CARD domain-containing protein 4
NLRP	NACHT, LRR, and PYD domains-containing protein
NLRs	Nucleotide-binding oligomerization domain and leucine-rich repeat-containing receptors
PAMPs	Pathogen-associated molecular pattern molecules
PET	Positron emission tomography
PI3K	Phosphatidylinositol 3-kinase
PPR	Pattern recognition receptor
PGE_2_	Prostaglandin E2
P2X4/P2RX7	Purinergic receptor subtypes
ROS	Reactive oxygen species
TLRs	Toll-like receptors
TSPO	Translocator protein
TSPO V_T_	Translocator protein total volume
VPL	Ventroposterior lateral thalamic nucleus
VPM	Ventroposterior medial thalamic nucleus
